# Reproductive health services utilization and its associated factors among Madawalabu University students, Southeast Ethiopia: cross-sectional study

**DOI:** 10.1186/s13104-015-0975-5

**Published:** 2015-01-17

**Authors:** Nagasa Dida, Birhanu Darega, Abulie Takele

**Affiliations:** Department of Public Health, College of Medicine and Health Sciences, Madawalabu University, P. O. Box: 302, Bale-Goba, Ethiopia; Department of Nursing, College of Medicine and Health Sciences, Madawalabu University, Bale-Robe, Ethiopia

**Keywords:** University students, Reproductive health services, Modern contraceptives, VCT and sexually transmitted infection diagnosis and treatment

## Abstract

**Background:**

Youths in Sub-Saharan region including Ethiopia account for higher proportion of new HIV infections, maternal mortality ratios, and unmet need for reproductive health information and services. This study assessed reproductive health services utilization and its associated factors among Madawalabu University Students, Southeast Ethiopia.

**Methods:**

Institutional-based cross-sectional study was conducted among regular under graduate Madawalabu University students in May 2014. Data were collected from randomly selected students through self-administered pre-tested structured questionnaire. Data were entered in to EpiData 3.1 and exported to SPSS-16.0 for analysis. Descriptive, bivariate and multivariate analyses were employed.

**Result:**

From the total 568 respondents 507(89.3%) of them knew modern family planning. 457(80.5%) of them had ever utilized at least one reproductive health services. Students who ever made discussion on VCT with health profession utilized the VCT two times than those hadn’t made discussion (AOR 2.06, 95% CI 1.21-3.48). Discussion also triple reproductive health services utilization (AOR 3.76, CI 1.55-9.11).

**Conclusion:**

Utilization of reproductive health services for the three indexed variables namely: modern contraceptives, STI diagnosis and treatment, and VCT is fair. But utilization of specific reproductive health services is under expectation. Discussion on reproductive health services between health worker and students, and focusing other identified factors are the way of reproductive health problems intervention in the University.

## Background

Reproductive health is a state of complete physical, mental and social well-being and not merely the absence of disease or infirmity, in all matters relating to the reproductive system and to its functions and processes. Reproductive health therefore implies that people are able to have a satisfying and safe sex life and that they have the capability to reproduce and the freedom to decide if, when and how often to do so [1].

Recently, youths number is estimated to be 1.25 billion globally [2] of which 85% of them is in developing countries [3] in which they are most vulnerable to a range of reproductive health problems, including too-early pregnancy and childbearing; unsafe abortion and sexually transmitted infections (STIs) including Human Immuno Deficiency Virus (HIV) [4].

Sub-Saharan Africa remains the most affected region in the world with an estimate of 22.5 million people living with HIV. Approximately 1.7 million new infections occurred in sub-Saharan Africa in the year 2007. Ten million young people aged 15–24 years and almost 3 million children under 15 years are living with HIV [5]. In Ethiopia young people age 10-24 constitute a third of the population. Premarital sexual activity is common and is on the rise worldwide. Rates are highest in sub-Saharan Africa, where more than half of girls aged 15-19 sexual experience because of which certain health problems are more prevalent in this age group [6]. According to the World Health Organization about one half of all HIV infections worldwide occur among people aged 25 years and under. And up to 100 million youths become infected with a curable sexually transmitted disease (STD); best estimates indicate that about one out of 20 youths in the world contract an STD each year [7]. Each day 500,000 young people are infected with an STI and in Africa the WHO estimates that 60% of all new HIV- infections occur in youths aged 15-19 years [8].

University students are the most vulnerable group for HIV infection due to their inclination to be engaged in risky sexual behavior and their sense of non-vulnerability [9]. Especially in countries with high prevalence of HIV/AIDS, significant proportion their students and staffs have been infected with HIV [10]. Voluntary Counseling and HIV testing is one of the main intervention strategies currently employed by National AIDS Commission. VCT provides an opportunity for a person to ascertain HIV status, and if infected with HIV, to prevent both transmission to others and re-infection. It also offers an opportunity to access care and support programs, including prophylaxis and treatment of opportunistic infections, access to antiretroviral therapy (ART) and access to PMTCT programs [11]. The college/university environment increases the susceptibility of its community for HIV infection [12] as a result of HIV high risk behaviors.

Furthermore, recent preliminary reports indicate that the risk behaviors among university students are increasing at an alarming rate. There are many risk factors fueling the spread of HIV and other communicable infections (e.g., sexually transmitted infections - STIs) among this group in particular. However, the response has been fragmented in higher education institutions (HEIs). Since the majority of Ethiopian universities have only recently been established, most university officials have not yet made HIV and STI prevention a priority and have little experience implementing HIV and STI prevention, despite these being environments that expose students to greater opportunities and circumstances that increase risky behavior [13]. Thus this study aimed to asses reproductive health service utilization specifically VCT, modern contraceptives and STI diagnosis and treatment service and its correlated factors to be used as preliminary data in Madawalabu University for HIV/AIDS prevention and control program.

## Methods

### Study setting and design

Institutional-based cross-sectional study was conducted among five hundred sixty eight randomly selected regular undergraduate students of Madawalabu University in May, 2014. Madawalabu University is one of the newly established Public universities in 2007. It found in Bale Zone, Oromia regional State, at a distance of 430 km away from Addis Ababa to Southeast of Ethiopia. The university has two campuses (the main campus in Robe town and Medicine and Health Sciences College campus in Goba town) with a total of 5,960 regular undergraduate students that run under one-college, one-institute and nine schools.

### Sampling and participation

Regular undergraduate students of Madawalabu University were the source population and the study population were those selected students for study through simple random sampling from the source population. The sample size was determined by using a single population proportion formula considering the assumptions: proportion of VCT service utilization which was 52.8% [14], level of confidence of 95% and margin of error 0.05.

The university was stratified into health and non-health school. From the total ten non-health schools and one medicine and health Sciences College of the university, eight schools/college were selected randomly. The total sample size of the study was allocated proportionally for the schools/college. Sample allocated for the schools/college were allocated proportionally for the stratified class year under the departments of selected schools/college. Finally, simple random sampling was employed to reach the study subject.

### Data collection tool and method

The questionnaire was adapted from different literature that was pertinent to the topic [13-18]. Data were collected through self-administered questionnaire. Supervision of the data collection was made by the investigators and supervisor. The data collection facilitators were from instructors from different schools. Data collection facilitators should be fluent of speakers of both Afan Oromo and Amharic language. Questionnaire was translated to the local language (Afan Oromo and Amharic) and translated back to English by different language experts. A two days training were given for data collector on data collection tool and procedure.

### Data management and analysis

Before data entry questionnaires were checked for completeness and double entry was made for 10% of the questionnaires using EpiData 3.1. and exported to SPSS windows version 16.0 for analysis. Descriptive analysis results were presented by tables. Bivariate logistic regression analysis was used to identify associations between variables. The possible effects of confounders were controlled through multivariate logistic regression analysis of backward conditional method with an entry of 0.05 and 0.1 removal. Association between the explanatory and dependent variable were assessed at p-value of 0.05.

### Ethical consideration

Ethical issues were approved by Madawalabu University Research Ethical Review Committee. Communication was made with schools before data collection. Prior to administering the questionnaires, the objectives of the study were clearly explained to the participants and oral informed consent was obtained. Confidentiality and anonymity were ensured throughout the execution of the study. Participants were informed that their participations were voluntary that they could withdraw from the study at any time if they wish to do so and this was not affect any service or benefit that they were get from any institution.

## Result

### Socio-demographic characteristics of the respondents

From the total 617 participants, 568 of them responded to the questionnaires that produce a response rate of 92.1%. The mean and median ages of the respondents were 21.3 (SD ± 1.68) and 21.00 respectively. More than half 347 (61.1%) of the respondents were rural in origin. Except a few, all respondents were living in the dormitory of the university. Two hundred twelve (37.3%) were Orthodox Christian followed by Muslim 163(28.7) in religion. Regarding their year of study 185(32.6%) were year one. Five hundred three (88.9%) of the respondents were single in their marital status (Table 1).

**Table 1:** **Socio-demographic characteristics of students of Madawalabu University, May, 2014**

**Variables**	**Frequency (N = 568)**	**Percent (%)**
**Campus of the University**		
Robe-main	480	84.5
Goba	88	15.5
**Origin of residence**		
Rural	347	61.1
Urban	221	38.9
**Present residence**		
In the university	562	98.9
Out of the university	6	1.1
**Class year**		
I	185	32.6
II	201	35.4
II	150	26.4
IV+	32	5.6
**Sex**		
Male	379	66.8
Female	188	33.2
**Marital status**		
Single	503	88.9
In plan of marriage	42	7.4
Married	18	3.2
Divorced	3	.5
**Religion**		
Orthodox	212	37.3
Muslim	163	28.7
Protestant	155	27.3
Wakefata	21	3.7
Other*	17	3
**Age**		
15-19	46	8.3
20-24	499	89.7
25+	11	2.0

*Catholic, Adventist, and other Christian.

### Utilization of family planning services

From the total 568 respondents, 536(94.4%) and 507(89.3) of them heard and know modern family planning respectively. Condom was known among 366 (72.2%) of the respondents as modern contraceptives. Pills, depo-provera, norplant and IUCD were also among the known contraceptives in their decreasing order by the respondents. Students’ clinic was the least known institution in providing modern contraceptives for the users. Twenty five (4.4%) of the respondents didn’t know where modern contraceptive present. Two hundred twenty nine (40.3%) of the respondents were sexually active. The mean and median ages of the respondents’ first sexual intercourse were 18.3 (SD ± 2.18) and 18 years respectively. 157(27.7%) of the respondents have ever used modern contraceptives. But from those sexually active respondents 96(42%) of them haven’t used any modern contraceptives ever and only one hundred eighteen (54%) of sexually active students used modern contraceptives for their last sex. Too young to start modern contraceptives 82 (51.2%), feel afraid 32 (20.1%) and religious opposition 28 (17.6%) were the major reasons for not utilizing contraceptives among sexually active respondents (Table 2).

**Table 2:** **Knowledge and utilization of modern contraceptives among students of Madawalabu University, May, 2014**

**Variables**			**Frequency**	**Percent**
**Know any modern contraceptive(s)**		Yes	507	89.3
		No	61	10.7
**Know - condom**		Yes	366	72.2
		No	141	27.8
**Know-pills**		Yes	314	61.9
		No	193	38.1
**Know-depo provera**		Yes	305	60.2
		No	202	39.8
**Know-norplant**		Yes	227	44.8
		No	280	55.2
**Know-IUCD**		Yes	185	36.5
		No	322	63.5
**Know - other**		Yes	30	5.9
		No	477	94.1
**Where family planning provided**	Health center	Yes	482	85.2
		No	84	14.8
	Hospital	Yes	312	55.1
		No	254	44.9
	Private clinic	Yes	187	33.0
		No	379	67.0
	Pharmacy	Yes	171	30.2
		No	395	69.8
	Student clinic	Yes	148	26.1
		No	418	73.9
	Other	Yes	24	4.2
		No	542	95.8
	Don’t know	Yes	25	4.4
		No	541	95.6
**What was the reason for not using contraceptives services**				
Too young to start contraception		Yes	82	51.2
		No	78	48.8
Feel afraid/embarrassed to get the service		Yes	32	20.1
		No	127	79.9
Religious opposition		Yes	28	17.6
		No	131	82.4
Don’t know how to use		Yes	11	6.9
		No	148	93.1
Fear of side effects		Yes	17	10.8
		No	140	89.2
Opposition from Sexual partner		Yes	14	8.8
		No	145	91.2
Lack of privacy by health workers		Yes	9	5.7
		No	148	94.3

### Voluntary counseling and testing of HIV/AIDS utilization

Majority (95.6%) of the respondents hear about voluntary counseling and testing of HIV/AIDS. Eighty two percent of the respondents had ever discussed over VCT. Only 50 (8.8%) of the respondents felt they were at risk of HIV. Among the total respondents of the study 421(74.1%) of them took voluntary counseling and testing of HIV/AIDS services. To know their sero status for HIV and order of health profession because of their diseases were the major reasons for VCT utilization among 382 (90.7%) and 22 (5.2%) respectively. Four hundred sixty six (82.3%) of the respondents prefer face to face provision of HIV test result. Perceiving not at risk of HIV/AIDS was the major reason for non-utilization of VCT service although 46 (31%) of them were sexually active (Table 3).

**Table 3:** **Voluntary counseling and testing of HIV/AIDS services utilization and related variables of students of Madawalabu University, May, 2014**

**Variables**		**Frequency**	**Percent**
Heard about VCT* service	Yes	542	95.6
	No	25	4.4
Discussed about VCT service	Yes	465	81.9
	No	103	18.1
Perceived at risk of HIV	Yes	50	8.8
	No	517	91.0
Used VCT service	Yes	421	74.1
	No	147	25.9
Major reason for utilization of VCT	To know my HIV sero status	382	90.7
	For marriage	14	3.3
	Order of health profession because of my disease	22	5.2
	Other	3	.7
Preferable ways of getting HIV test result	Face to face	466	82.3
	With secret letter	94	16.6
	Other	6	1.1

*VCT-voluntary counseling and testing.

### VCT and associated factors

Using bivariate logistic regression, factors associated with voluntary counseling and testing of HIV/AIDS were identified. Sexual intercourse history, history of STI syndrome, knowing hospital provided VCT services and having discussion on VCT with profession showed statistically significant association with voluntary counseling and testing uptake. However, history of STI symptom, history of discussion on VCT with health profession and hearing VCT information from health profession found to be the predictors of voluntary counseling and testing uptake. Those students who ever made discussion on VCT with the health profession made them two fold to take VCT [OR (95% CI), 2.06 (1.21-3.48)] (Table 4).

**Table 4:** **Factors associated with VCT among Madawalabu University students, May, 2014**

**Factors**		**VCT use**		**COR [95% C.I], P-value**	**AOR [95% C.I]**
		**Yes**	**No**		
Had sexual intercourse history	Yes	183	46	1.67 (1.13-2.510), 0.01	0.99 (0.60-1.63)
	No	238	101	1.00	1.00
Experienced STI syndrome	Yes	104	25	1.60 (1.01-2.60), 0.04	1.99 (1.04-3.82)*
	No	317	122		1.00
Discussed about VCT service	Yes	379	86	6.40 (4.05-10.11), 0.00	4.19 (1.68-10.45)
	No	42	61	1.00	1.00
Know any modern contraceptive	Yes	385	122	2.19 (1.27-3.80), 0.00	0.96 (0.39-2.6)
	No	36	25	1.00	1.00
Ever discussed VCT with Health profession	Yes	185	28	1.98 (1.21-3.23), 0.00	2.06 (1.21-3.48)*
	No	194	58	1.00	1.00
Knowing that hospital provides VCT service	Yes	298	87	1.67 (1.13-2.47), 0.01	1.31 (0.78-2.23)
	No	123	60	1.00	1.00
Health profession as a source of information of VCT	Yes	270	69	1.91 (1.29-2.84), 0.00	1.62 (1.01-2.69)*
	No	137	67	1.00	1.00

*P-value < 0.05.

### Reproductive health service utilization and associated factor

From the total 568 respondents of the study, 457 (80.5%) ever utilized reproductive health services. The three reproductive health components addressed in this study namely, modern contraceptive, STI diagnosis and treatment and VCT were indexed for their utilization. Then factors associated with the utilization of reproductive health were identified using bivariate logistic regression. Sexual intercourse history, ever discussed on the three indexed variables, knowledge of modern contraceptive, history of STI syndrome and class year of the respondents were independently associated with reproductive health service utilization. When variables associated under bivariate logistic regression adjusted for possible confounding factors through multivariate logistic regression, sexually active, knowing any type of modern contraceptive, discussion on family planning with health profession and making discussion of VCT were found determinant factors of reproductive health service utilization. Sexually active respondents utilized reproductive health service six time more than sexually inactive students [AOR (95% C.I), 5.99 (2.60-13.81)]. Second year students were 0.30 less likely to utilize reproductive health service when compared with year one respondent [AOR (95%, CI); 0.30 (0.13-0.70)] (Table 5).

**Table 5:** **Factors associated with reproductive health service utilization among Madawalabu University students, May, 2014**

**Factors**		**RH use**		**COR [95% C.I], P-value**	**AOR [95% C.I]**
		**Yes**	**No**		
Had history sexual intercourse	Yes	212	17	4.79 (2.77-8.28), 0.00	5.99 (2.60-13.81)*
	No	245	94	1.00	1.00
Know any modern contraceptive	Yes	419	88	2.88 (1.64-5.08), 0.00	3.55 (1.26-9.99)*
	No	38	23	1.00	100
Ever discussed on modern contraceptives	Yes	339	59	2.53 (1.65-3.88), 0.00	-
	No	118	52	1.00	-
Discussion on family planning with partner	Yes	144	14	2.40 (1.27-4.54), 0.00	1.37 (0.64-2.91)
	No	193	45		
Discussion on family planning with health profession	Yes	92	6	3.32 (1.38-7.98), 0.007	2.90 (1.06-7.96)*
	No	245	53	1.00	1.00
Had STI diagnosis and treatment service information	Yes	418	91	2.36 (1.31-4.23), 0.004	-
	No	39	20	1.00	-
Health profession as STI diagnosis & treatment service information source	Yes	238	40	1.69 (1.07-2.66), 0.025	1.13 (0.57-2.21)
	No	180	51	1.00	1.00
Discussed about STI	Yes	284	52	1.86 (1.23-2.83), 0.004	1.18 (0.58-2.42)
	No	173	59	1.00	1.00
Heard VCT service	Yes	441	101	2.91 (1.27-6.67), 0.012	0.78 (0.14-4.40)
	No	15	10	1.00	1.00
Discussed on VCT	Yes	406	59	7.02 (4.37-11.26), 0.000	3.76 (1.55-9.11)*
	No	51	52	1.00	1.00
Experienced STI syndrome	Yes	116	13	2.56 (1.39-4.75), 0.003	1.54 (0.67-3.51)
	No	341	98	1.00	1.00
Class year	I	133	52	1.00	1.00
	II	174	27	0.40 (0.24-0.67), 0.000	0.30 (0.13-0.70)*
	III	124	26	0.54 (0.32-0.91), 0.021	0.56 (0.25-1.24)
	IV+	26	6	0.59 (0.23-0.1.52), 0.23	1.08 (0.31-3.78)

*P-value < 0.01.

## Discussion

Two hundred twenty nine (40.3%) of the respondents were sexually active. This result was slightly higher and lower than students of Wolayita Sodo University and Addis Ababa University (AAU). In Wolayita Sodo University 158(35.3%) of the students were sexually active where as fifty percent of AAU students were sexually active [13,19]. The mean of the respondents’ first sexual intercourse was 18.3 (SD ± 2.18) years which was nearly similar with students of Wolayita Sodo University – 17.7 (SD ± 4.9) years [19].

From the total 568 respondents 507 (89.3%) of them knew modern contraceptives which was higher than female students of Hawassa University where four hundred ninety one (63.3%) of the students had knowledge about modern contraceptives. Three hundred sixty six (72.2%) of the respondents knew condom as one of the modern contraceptives. Pills, depo-provera, norplant and IUCD were known modern contraceptives in their decreasing order by the respondents. This is different from the findings of Hawassa University where the most commonly known contraceptives were 301 (38%) pills, 110 (14.2%) condom and 51 (7%) injectiables. This might be because differences of composition of the study population [16]. Among East Gojjam youth also knowledge about reproductive health was lower than this study finding [20].

One hundred fifty seven (27.7%) of the total respondents ever used contraceptives. This is lower than study done among female Hawasa University which was 328 (67%) of the students ever used modern contraceptives [16]. From those sexually active respondents similar figure 133 (58%) ever used any form of modern contraceptives and half of those sexually active students of AAU also use condoms [13]. This is higher than sexually active youths of Vietnamese college students, which was only 32% of females and 28% of males who ever used a contraceptive method at first sexual intercourse [21]. This difference might be because of socio-demographic difference and description of the variables for both sex in the case of Vietnamese. In Dakar young adults also contraceptive use at time of first sexual experience was less which was 30% [22]. Among unmarried sexually active youths in Sub- Saharan Africa, contraceptive use ranges from of 3% in Rwanda to 56% in Burkina Faso [23]. This study also lies in the same range.

Majority (95.6%) of the respondents heard about voluntary counseling and testing of HIV/AIDS which was higher than Debre Markos University Students where 579 (81.4%) of students heard the presence of VCT service [17]. Among the total respondents of the study 421 (74.1%) of them took voluntary counseling and testing of HIV/AIDS services which was higher than study conducted among Harar College students where only 52.8% of the respondents have been tested for HIV. Student who had been tested for HIV infection among college of nursing students in Malawi was 68% which was slightly lower than the case of this study [18]. The major reasons for their VCT utilization was to know their HIV sero status 382 (90.7%) and order of health professionals because of their diseases 22 (5.2%). Among Harar college Ethiopia students 89.9% and 3.8% them took VCT to know their HIV sero status and to engage in marriage respectively which is similar to this study [14]. Perceiving that they were not at risk of HIV/AIDS was the major reason for non-utilization which was also the main reason for Debre Markos University Students [17].

History of discussion on VCT with health professionals and hearing VCT information from health professionals become the predictors of voluntary counseling and testing service uptake. Those students who ever made discussion on VCT with the health professionals made them two fold likely to take VCT [OR (95% CI), 2.06 (1.21-3.48)]. Among Jamaican university students attending an HIV education forum increased likelihood of previous HIV test report [24,25] identified, there was an association between knowing where to get tested and taking an HIV test (p = 0.003). Religion was also associated with having taken an HIV test (p = 0.027) which was different from the finding of this study.

Sexual status, knowing any type of modern contraceptive, discussion on family planning with health professionals and making discussion of VCT were the determinant factors for reproductive health service utilization. Sexually active respondents utilized reproductive health service six time more than sexually inactive students [AOR (95% C.I), 5.99 (2.60-13.81)]. Study conducted in Jimma also identified sexual activity was the determinant factors of reproductive health service utilization [26]. But age and marital status which were significantly associated in study conducted in Jimma and Jamaican students were insignificant in this study [24,26]. Youths who had interaction with family and peers and had access to pamphlets and posters as source of information for RH services were more likely to be ever used reproductive health services [26].

This study can be considered with certain limitations. As data were collected through self administered for self reporting there might be under or lower reporting. Data collection period which was during an exam period, when most of the students were in stress might make the students to rash over some variables.

## Conclusion

Utilization of reproductive health services for the three indexed services namely: modern contraceptives, STI diagnosis and treatment and VCT is fair. But utilization of specific reproductive health services is under expectation. Discussion of reproductive health services especially with health worker, class year of the respondents were among the most determinant factors of reproductive health service utilization. There were students who were sexually active and were not aware for their risk for HIV/AIDS. Discussion of reproductive health service is valuable for the utilization of the services by the students. Strengthening reproductive health services discussion with the students and focusing other identified factors for an intervention of reproductive health problems is valuable for the University students.
